# Hydroxysafflor yellow A, a natural food pigment, ameliorates atherosclerosis in ApoE ^−/−^ mice by inhibiting the SphK1/S1P/S1PR3 pathway

**DOI:** 10.1002/fsn3.4466

**Published:** 2024-09-14

**Authors:** Yuqing Liu, Jingyu Piao, Jiqian He, Zhuoxuan Su, Zhizhong Luo, Duosheng Luo

**Affiliations:** ^1^ Guangdong Metabolic Diseases Research Center of Integrated Chinese and Western Medicine, Key Laboratory of Glucolipid Metabolic Disorder, Ministry of Education of China, Guangdong Key Laboratory of Metabolic Disease Prevention and Treatment of Traditional Chinese Medicine, Institute of Chinese Medicine Guangdong Pharmaceutical University Guangzhou Guangdong China

**Keywords:** atherosclerosis, hydroxysafflor yellow A, SphK1/S1P/S1PR3 pathway, vascular permeability

## Abstract

Atherosclerosis (AS) is the pathologic basis of many cardiovascular diseases (CVDs). Hydroxysafflor yellow A (HSYA) is a valuable natural food pigment that has been reported to have significant health‐promoting abilities. However, the anti‐AS efficacy and mechanisms of HSYA have not yet been characterized. Here, we found that treatment of apolipoprotein A (ApoE) knockout (ApoE^−/−^) mice with HSYA markedly ameliorated atherosclerosis evidenced by decreased levels of lipids, sphingosine‐1‐phosphate (S1P), inflammatory factors, oxidative stress, vascular endothelial permeability, and endothelial damage. Moreover, mechanistic studies revealed that HSYA treatment downregulated the expression of aortic sphingosine kinase 1 (SphK1), sphingosine‐1‐phosphate receptor 3 (S1PR3), Ras homolog family member A (RhoA), Rho‐associated coiled‐coil containing protein kinase (ROCK), and filamentous actin (F‐actin). The results of administration with HSYA reversed the effects of SphK1 agonist and S1PR3 agonist on oxidized low‐density lipoprotein (ox‐LDL)‐induced vascular endothelial cell migration ability and F‐actin expression, and decreased RhoA/ROCK protein expression further confirmed the conclusion that HSYA reduced vascular endothelial permeability by modulating the SphK1/S1P/S1PR3/RhoA/ROCK signaling pathway, thereby exerting anti‐atherosclerotic effects. Overall, this study indicated that HSYA might be a therapeutic candidate for the treatment of AS.

## Graphical abstract

Natural food pigment hydroxysafflor yellow A (HSYA) has therapeutic effects on atherosclerosis (AS). Hydroxysafflor yellow A (HSYA) improved lipid, inflammation, oxidative stress, endothelial dysfunction and alleviated aortic lesions in AS mice. Hydroxysafflor yellow A (HSYA) attenuated aortic endothelial permeability by inhibiting the sphingosine kinase 1 (SphK1)/sphingosine‐1‐phosphate (S1P)/sphingosine‐1‐phosphate receptor 3 (S1PR3) pathway.

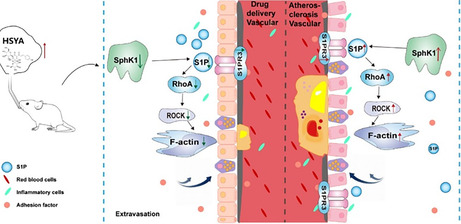

## 1 INTRODUCTION

Atherosclerosis (AS) leads to the development of myocardial ischemia as well as a range of cardiovascular diseases (CVDs), such as stroke and peripheral vascular disease (PVD). According to the American Heart Association (AHA), the number of people with CVD worldwide has totaled 19 million since 2010 (Tsao et al., 2022). About 7.6 million people die of CVD in China, accounting for about 40% of global CVD deaths (Wang et al., 2023). And one of the keys to the development of AS is vascular endothelial dysfunction (Akhtar & Sharma, 2022). Vascular oxidative stress, inflammation, and increased endothelial permeability are all factors that can induce endothelial cell dysfunction (Diao et al., 2022). Thus, protection of endothelial cells can effectively delay and block the pathologic process of AS (Zhu et al., 2018).

An increasing number of studies have shown that many natural medicines have significant efficacy in the treatment of AS (Duan et al., 2021). Hydroxysafflor yellow A (HSYA) is a natural and safe food pigment, widely found in edible plants, such as *Carthamus tinctorius* L., which belongs to flavonoids with anti‐inflammatory, antioxidant, blood pressure‐lowering, antimyocardial ischemia, and coronary artery vasodilating effects. Studies have shown that HSYA reduces the expression of aortic inflammatory factors and adhesion molecules, as well as lowering lipids and inhibiting oxidative stress (Hu et al., 2020; Zhou et al., 2018). Furthermore, HSYA improves brain endothelial cell permeability and attenuates high glucose‐induced endothelial cell dysfunction in human umbilical veins (Chen et al., 2019; Sun et al., 2018; Yang et al., 2018). Given the endothelial protective effects of HSYA in other models and the characteristics of endothelial dysfunction in AS, it is of great significance to conduct in‐depth studies on the impact of HSYA on endothelial permeability in AS. Our previous study found that the deficiency of sphingosine kinase 1 (SphK1) attenuated vascular endothelial barrier function in AS through the SphK1/ sphingosine‐1‐phosphate (S1P)/sphingosine‐1‐phosphate receptor (S1PR) signaling pathway (Piao et al., 2024). However, whether HSYA ameliorates AS by modulating SphK1 to reduce vascular endothelial permeability is unclear.

In this study, we explored the treatment effect of HSYA on AS and clarified the relationship between HSYA and the SphK1/S1P/S1PR3 (sphingosine‐1‐phosphate receptor 3) signaling pathway in AS. These results might provide a scientific basis for the clinical application of HSYA in AS treatment and a potential clinical drug for AS.

## 2 MATERIALS AND METHODS

### 2.1 Drugs and reagents

Hydroxysafflor yellow A (HSYA) (CAS: 78281‐02‐4, HPLC ≥90%) was purchased from Sichuan Vicci Biotechnology Co., Ltd. (Chengdu, China). Simvastatin was purchased from Merck Sharp & Dohme Limited (United Kingdom). The high fat diet (HFD, 60% fat, 20% protein, and 20% carbohydrates) was obtained from Dyets Biotechnology Co., Ltd. (USA). High‐density lipoprotein cholesterol (HDL‐C), low‐density lipoprotein cholesterol (LDL‐C), triglyceride (TG), total cholesterol (TC), superoxide dismutase (SOD), and malondialdehyde (MDA) assay kits were purchased from Jiancheng Biotechnology Co., Ltd. (Nanjing, China). Interleukin‐6 (IL‐6), interleukin 1‐beta (IL‐1β), tumor necrosis factor‐alpha (TNF‐α), S1P, and endothelin‐1 (ET‐1) assay kits were purchased from MEIMIAN Biological Co., Ltd. (Jiangsu, China). Nitric oxide (NO) kit was purchased from Meilun Biological Co., Ltd. (Dalian, China).

### 2.2 Animal models establishment and drug administration

Eight‐week‐old C57BL/6 male mice and apolipoprotein A (ApoE^−/−^) mice were obtained from Vital River Laboratory Animal Technology Co., Ltd. (Beijing, China). The animals were raised under standard environmental conditions with relative humidity (RH) at 40%–70% and temperature at 20°C–26°C. All animal experiments were approved by the Laboratory Animal Management and Ethics Committee of Guangdong Pharmaceutical University (GDPULAC2020059, Guangzhou, China). In this study, the animal experiments followed the Ethics Committee of Guangdong Pharmaceutical University and the existing current animal welfare guidelines.

The mice were fed an atherogenic HFD (60% fat, 20% protein, and 20% carbohydrates) for 12 weeks (Piao et al., 2022). Between the groups of mice, there was no significant difference in the initial basal body weight. Mice were randomized into five groups: Control group (Ctrl, *n* = 6), Model group (Mode, *n* = 6), HSYA high dose group (HSYA‐H, 200 mg/kg/day (Lee et al., 2020; Liu et al., 2018), *n* = 6), HSYA low dose group (HSYA‐L, 100 mg/kg/day, *n* = 6), and Simvastatin group (Sim, 5 mg/kg/day (Sozański et al., 2014), *n* = 6). All the above five groups were fed with HFD for 12 weeks. The HSYA and Sim groups were given a gavage of the respective drugs dissolved in 0.9% saline on a daily basis according to body weight. The Model and Ctrl groups were gavaged daily with saline. After 12 weeks of intervention, isoflurane anesthesia after 12 h of fasting, blood was collected by eyeball blood sampling. The aorta and heart were collected and stored at −80°C for subsequent use.

### 2.3 Analysis of body composition

Lean and fat mass were determined by the EchoMRI body composition analyzer (EchoMRI™, Shanghai, China) in mice, according to the manufacturer's instructions. Body fat percentage per mice = fat mass/body weight (g/g) × 100%.

### 2.4 Measurement of biochemical parameters in serum

Blood samples were centrifuged at 3000 rpm (revolutions per minute) for 15 min to obtain serum. The levels of TC, TG, LDL‐C, and HDL‐C were assayed using biochemical parameter test kits. In addition, serum was used to measure the level of IL‐6, S1P, IL‐1β, TNF‐α, ET‐1, and NO by enzyme‐linked immunosorbent assay (ELISA) kits. All methods were performed, according to the manufacturer's instructions.

### 2.5 Histopathological analysis

The aortic root was fixed with 4% paraformaldehyde (PFA), embedded in the optimal cutting temperature (OCT) medium, and transected at a thickness of 10 μm on a freezing sliding microtome. Lipid deposition was detected on the cross‐sections by Oil Red O staining (Yuanye, Shanghai, China). Hematoxylin and eosin (H&E) staining (Pinuofei, Wuhan, China) was used to quantify the size of atherosclerotic plaques. Sirius scarlet staining (Pinuofei, Wuhan, China) was performed to determine collagen fibers. Verhoeff–van Gieson (VVG) stain was used for elastic fibers. Images were captured with an optical microscope (Olympus, Japan), and data were processed with ImageJ software.

### 2.6 Immunohistochemical analysis

Arteries were fixed in 4% paraformaldehyde solution and sectioned by paraffin embedding. Arterial sections were incubated with 3% hydrogen peroxide (H_2_O_2_) for 25 min to inhibit endogenous peroxidase activity and then rinsed with phosphate‐buffered saline (PBS) for 15 min. Sections were blocked with 3% bovine serum albumin (BSA) for 30 min. Sections were incubated with vascular cell adhesion molecule 1 (VCAM‐1) anti‐rabbit antibody (Beyotime, Shanghai, China, dilution: 1:400) overnight at 4°C and washed for 15 min. Sections were then incubated with secondary antibody for 60 min at room temperature. Sections were stained with hematoxylin solution after color development with 3,3'‐diaminobenzidine (DAB) color solution and mounted with neutral balsam and coverslips. Photographs were taken with a light microscope and the area of positive staining was measured using ImageJ software.

### 2.7 Terminal deoxynucleotidyl transferase‐mediated dUTP nick end labeling assay

Tissues were fixed for at least 24 h in 4% paraformaldehyde, dehydrated in graded ethanol, and embedded in paraffin. Paraffin sections (5 μm) were incubated in terminal deoxynucleotidyl transferase‐mediated dUTP nick end labeling (TUNEL) reaction mixture from a kit (Meilun Biological Co., Ltd., Dalian, China), according to the manufacturer's instructions. Slides were scanned using a digital microscopy scanner.

### 2.8 Evans blue assay

One hour before execution, mice were injected with 0.5% 2 mL/kg Evans blue staining solution (Macklin, Shanghai, China) in the tail vein. Mice were anesthetized after the eyes appeared blue, and blood was removed from the eyeballs for neck dislocation and execution. The thorax and abdomen of the mice were dissected, excess fatty tissue and organs were removed, and the aorta was separated with ophthalmic scissors and placed under the body microscope for observation and photographing. Then the arterial tissue was cut, homogenized and the supernatant was taken, added with trichloroacetic acid (TCA), put into the refrigerator at 4°C, and set aside. Add different concentrations of Evans blue staining solution to the 96‐well plate, and the rest of the wells were added with the supernatant that had been prepared, and incubated in the incubator at 37°C for 20 min, and the optical density (OD) value at 620 nm was measured by an enzyme labeling instrument, and the standard curve was plotted, according to the different concentrations of Evans blue staining solution, and the Evans blue content of the samples was calculated.

### 2.9 Cell culture and HSYA treatment

Human umbilical vein endothelial cells (HUVECs, enzyme‐linked, Shanghai, China) were cultured at 37°C and in a humidified atmosphere containing 5% carbon dioxide (CO_2_). Adherent cells were subcultured 2–3 times per week. The growth media for HUVECs were supplemented with 10% fetal bovine serum (FBS) (Pricella, Wuhan, China), 100 U/mL penicillin and 100 μg/mL streptomycin (Yuanye, Shanghai, China). The inflammatory response of vascular endothelial cells mediated by ox‐LDL is the early behavior and main signal of atherosclerosis (Mehta & Malik, 2006). Therefore, we used ox‐LDL (100 μg/mL, Yiyuan Biotechnology, Shanghai, China) (Zhang et al., 2024) to stimulate HUVECs for 24 h as an in vitro model of vascular endothelial hyperpermeability in AS. To investigate the effect of HSYA on SphK/S1P/S1PR‐mediated vascular endothelial permeability, cells were stimulated with the SphK1 agonist K6PC‐5 (10 μM, Monmouth Junction, NJ, USA) (Shao et al., 2015), the S1P agonist FTY‐720 (50 ng/mL, Monmouth Junction, NJ, USA) (Milford et al., 2022), or the S1PR3 agonist CYM5541 (100 nM, Monmouth Junction, NJ, USA) (Ziegler et al., 2024).

### 2.10 Cell counting kit‐8 assay

Cell viability was evaluated utilizing a Cell Counting Kit‐8 assay kit (CCK‐8; Yeasen Biotechnology, Shanghai, China) with a seeding density of 2 × 10^3^ cells per well in 96‐well plates. HUVECs were exposed to various concentrations of HSYA (0, 10, 20, 50, 100, 150, and 200 μM) for 24 h. The absorbance was then quantified using a microplate reader.

### 2.11 Wound‐healing assay

Human umbilical vein endothelial cells (HUVECs) were deposited in 6‐well plates at a density of 5 × 10^5^ per well. Scratch lines were created with a 200 μL pipette tip. The scratch lines were observed with a microscope at 24 h of incubation. The area of the original and final scratch lines was measured using ImageJ software.

### 2.12 Immunofluorescence staining

Human umbilical vein endothelial cells (HUVECs) were cultured in 6‐well plates. Different groups of HUVECs were treated with HSYA or ox‐LDL for 12 h and fixed with 4% PFA for 15 min. Cells were permeabilized with 0.1% Triton X‐100 followed by reaction with 2% BSA for 1 h. Cells were incubated overnight at 4°C with first antibody. On the following day, the specific secondary antibody was added and the samples were incubated for 1 h at room temperature after a wash in PBS.

### 2.13 Western blotting

Total proteins were extracted from the treated cells and aorta tissue by loading buffer. After measuring the concentration, the proteins were separated by sodium dodecyl sulfate‐polyacrylamide gel electrophoresis (SDS‐PAGE) and transferred to polyvinylidene fluoride (PVDF) membranes. Membranes were blocked with 5% bovine serum albumin (BSA) in Tris‐buffered saline Tween (TBST) solution for 1 h at 37°C, with the corresponding primary antibody (1:1000 dilution) overnight at 4°C and with the corresponding secondary antibody for 1 h at 37°C with Super Enhanced Chemiluminescence (ECL) reaction reagent (GBCBIO Technologies Inc.). SphK1, sphingosine kinase 2 (SphK2), and sphingosine‐1‐phosphate receptor 1 (S1PR1) anti‐rabbit antibodies were purchased from Proteintech Group, Inc. (Wuhan, China). S1PR3 antirabbit antibody from Absin Biological Technology Co., Ltd. (Shanghai, China). RhoA, ROCK antirabbit antibody, and glyceraldehyde‐3‐phosphate dehydrogenase (GAPDH) were purchased from Cell Signaling Technology, Inc. (Boston, USA). F‐actin anti‐rabbit antibody was obtained from Abcam (Shanghai) Trading Co., Ltd. (Shanghai, China). Goat anti‐Mouse Immunoglobulin G (IgG) Secondary Antibody Horseradish Peroxidase (HRP) and Goat anti‐Rabbit Immunoglobulin G (IgG) Secondary Antibody Horseradish Peroxidase (HRP) were purchased from Signalway Antibody (Nanjing, Jiangsu, China).

### 2.14 Statistical analysis

All results were showed as the mean ± standard error (SEM). Statistical analysis was conducted using the one‐way analysis of variance (ANOVA) with Tukey's post hoc test. A value of *p* < .05 was considered statistically significant. All data were performed using GraphPad Prism 8.0.

## 3 RESULTS

### 3.1 HSYA reduced body weight and blood lipids in ApoE ^−/−^ mice

**FIGURE 1:**
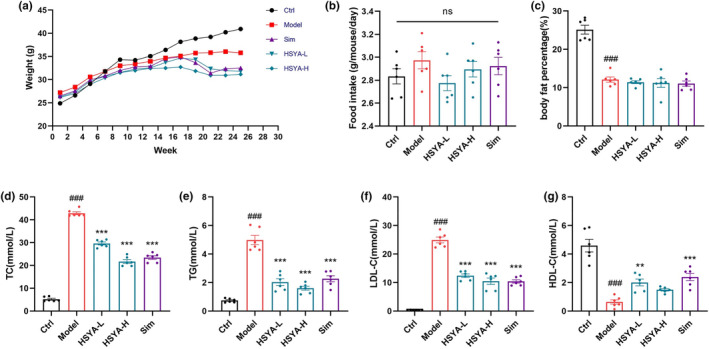
Hydroxysafflor yellow A (HSYA) reduced body weight and blood lipids in ApoE^−/−^ mice. (a) Body weight. (b) Food intake. (c) Body fat percentage, body fat percentage = fat mass/body weight (g/g) × 100%. (d–g) TC, TG, LDL‐C, and HDL‐C levels in serum were analyzed. Data were presented as the mean ± SEM (*n* = 6). ^###^ *p* < .001 versus Ctrl group; **p < .01, ***p < .001 versus Model group.

To determine the effects of HSYA on AS in ApoE^−/−^ mice, as displayed in the supporting data (Figure 1A–C), HSYA administration markedly decreased the body weight without affecting the food intake of AS mice. In addition, HSYA reduced the elevation of serum TC, TG, and LDL‐C (Figure 1D–F). Moreover, HSYA increased the degradation of serum HDL‐C (Figure 1G).

### 3.2 HSYA attenuated inflammation, oxidative stress, and endothelial damage in ApoE ^−/−^ mice

**FIGURE 2:**
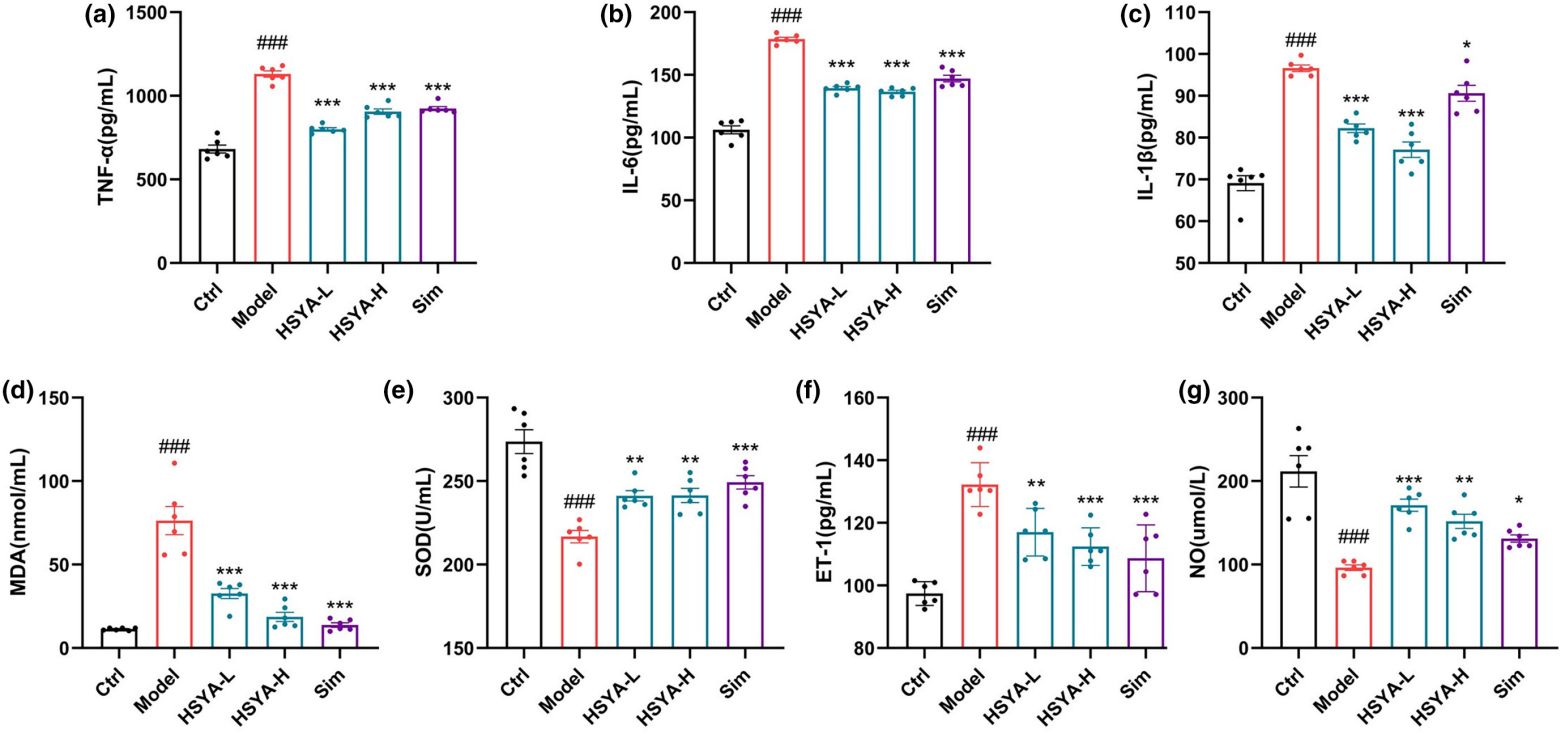
Hydroxysafflor yellow A (HSYA) attenuated inflammation, oxidative stress, and endothelial damage in ApoE^−/−^ mice. (a–c) Serum inflammatory factor levels. (d) Serum MDA content. (e) Serum SOD activity. (f) Serum ET‐1 levels. (g) Serum NO levels. Data were presented as the mean ± SEM (*n* = 6). ^###^ *p* < .001 versus Ctrl group; **p < .01, ***p < .001 versus Model group.

Treatment with HSYA significantly reduced serum TNF‐α, IL‐6, and IL‐1β (Figure 2A–C). As widely acknowledged, nuclear factor kappa B (NF‐κB) regulates the expression of a large number of genes involved in the initiation and development of atherosclerotic plaques, including cytokines (TNF‐α, IL‐1β, and IL‐6), adhesion molecules (VCAM‐1 and E‐selectin), etc., which increases the interaction of inflammatory cells, such as endothelial cells and leukocytes, and promotes inflammatory responses within atherosclerotic plaques (Lv et al., 2020; Milstone et al., 2015). Meanwhile, HSYA treatment significantly reduced MDA and increased SOD (Figure 2D,E). Oxidative stress leads to the oxidation of tetrahydrobiopterin (BH4), endothelial nitric oxide synthase (eNOS) uncoupling inactivation and decreased endothelial NO production, which results in impaired endothelium‐dependent diastolic function and induces endothelial cell dysfunction (Greaney et al., 2019). In addition, HSYA administration resulted in a significant decrease in serum ET‐1 levels and a significant increase in NO levels in mice (Figure 2G). Decreased NO bioavailability, vascular oxidative stress, and inflammation are all factors that can induce endothelial cell dysfunction. NO is a crucial endothelium‐derived relaxing factor that can regulate the vascular tone, maintaining vasodilatory and contractile balance (Förstermann et al., 2017). The above results showed that HSYA supplementation protected the vascular endothelium and attenuated endothelial injury in ApoE^−/−^ mice.

### 3.3 HSYA ameliorated aortic plaque area, lipid deposition, and aortic lesions in ApoE ^−/−^ mice

**FIGURE 3:**
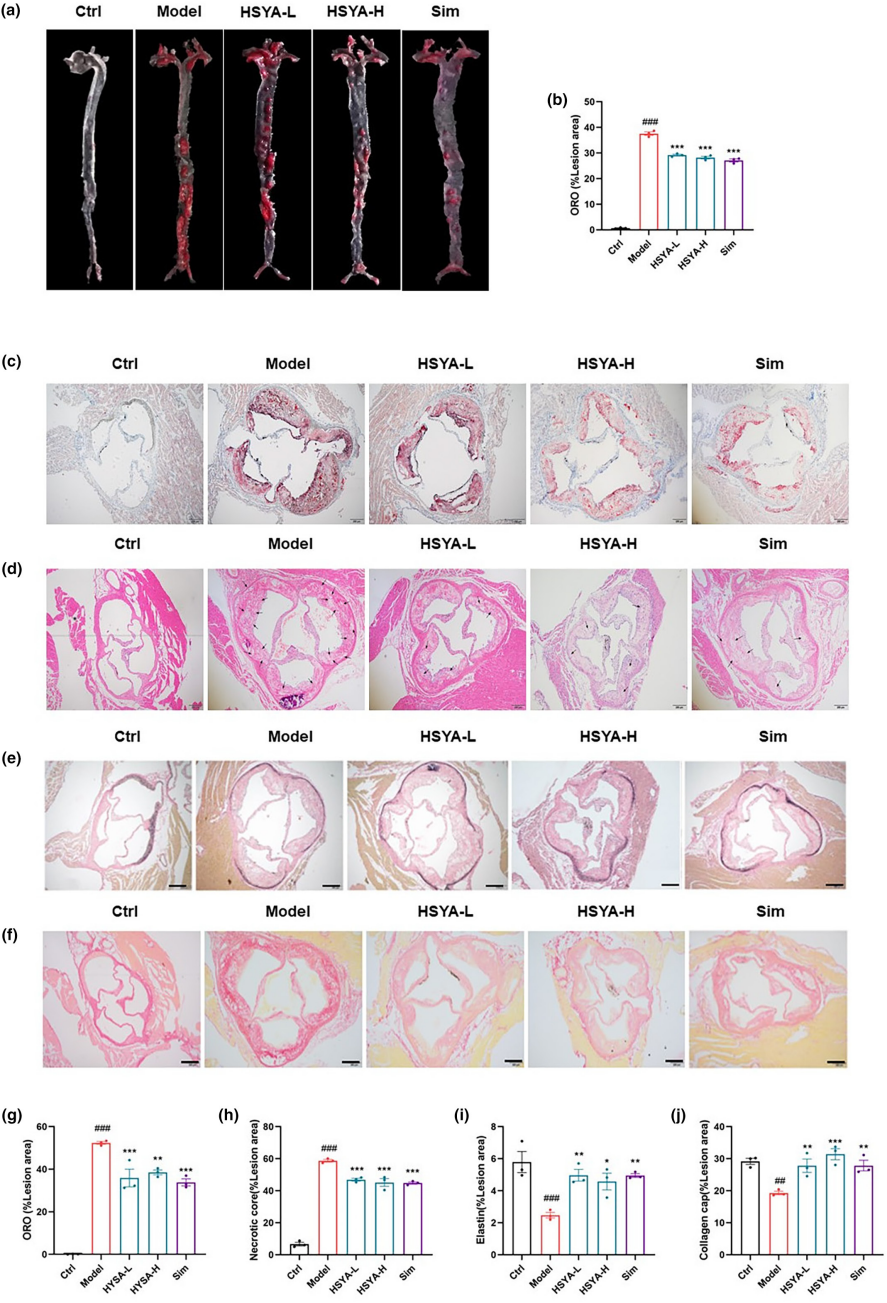
Hydroxysafflor yellow A (HSYA) ameliorated aortic plaque area, lipid deposition, and aortic lesions in ApoE^−/−^ mice. (a, b) Images and quantification of oil red O staining of whole aorta. Scale bar: 200 μm. (c, g) Quantification of cross‐section and plaque area of aortic root stained with oil red O. Scale bar: 200 μm. (d, h) Representative images of atherosclerotic lesions quantified by H&E staining and plaque size and lesion area as a percentage of aortic root area. Scale bar: 200 μm. (e, i) Representative images of EVG staining of the aortic root and the percentage of lesion area in the aortic root area. Scale bar: 200 μm. (f, j) Representative images of Sirius scarlet staining of the aortic root and the percentage of lesion area in the aortic root area. Scale bar: 200 μm. Data were presented as the mean ± SEM (*n* = 3). ^###^ *p* < .001 versus Ctrl group; *p < .05, **p < .01, ***p < .001 versus Model group.

Oil red O staining revealed that treatment with HSYA resulted in a noteworthy reduction of lipid droplets on the vascular wall of the aortic root and arterial plaque area (Figure 3A–C,G). H&E staining of the aortic root showed a distinct core area of necrosis in the aortic root in the Model group, with obvious inflammatory infiltration, which was repaired in the HSYA group (Figure 3D,H). We also conducted EVG and Sirius scarlet staining on the aortic root to explore the aortic lesions in ApoE^−/−^ mice and found that mice in the HSYA group had an increase in elastic fiber content, which contrasted with the Model group (Figure 3E,F,I,J). The above results indicated that HSYA has protective effects on ApoE^−/−^ mice.

### 3.4 HSYA enhanced vascular plaque stability and alleviated aortic endothelial permeability in ApoE ^−/−^ mice

**FIGURE 4:**
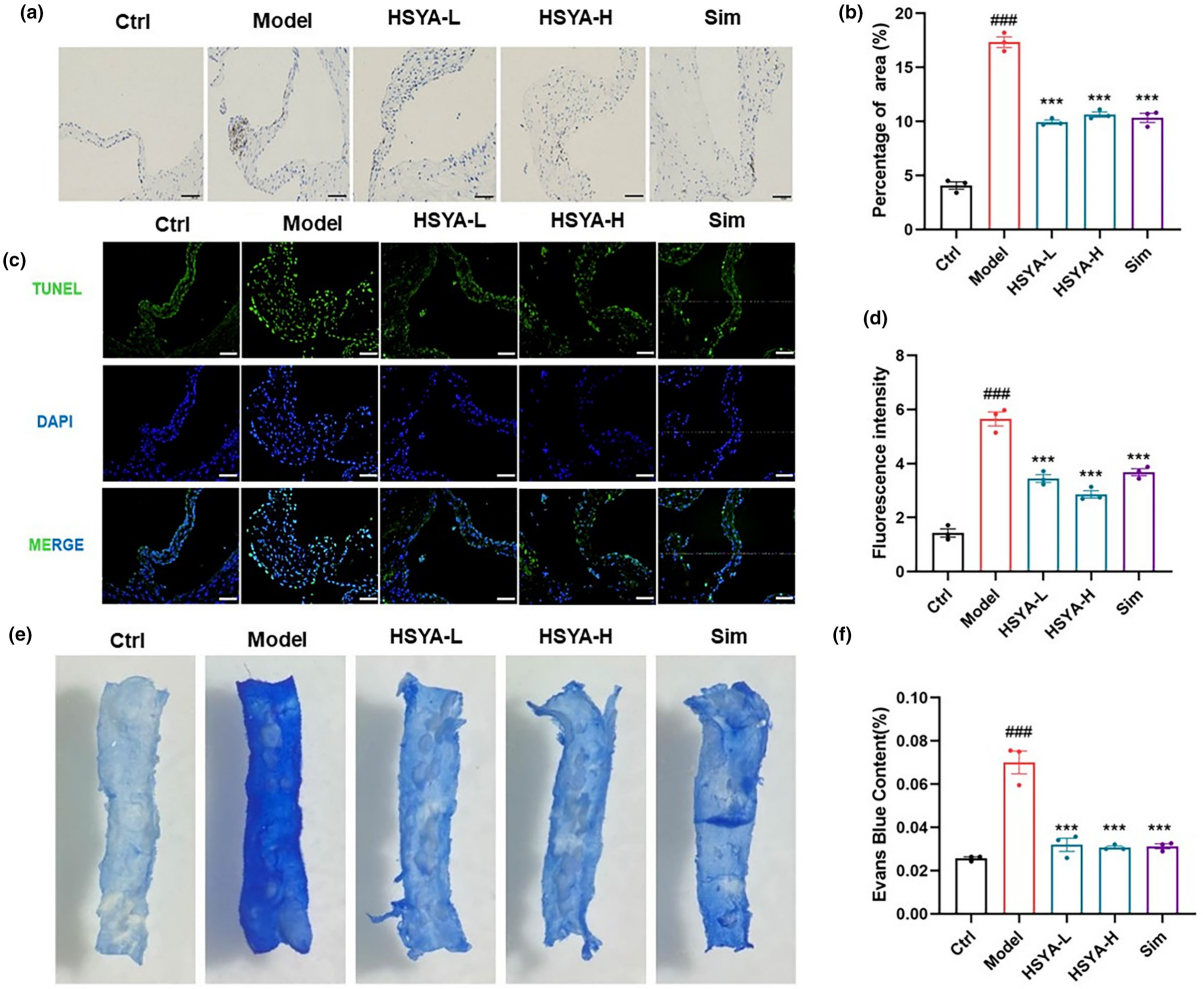
Hydroxysafflor yellow A (HSYA) enhanced vascular plaque stability and alleviated aortic endothelial permeability in ApoE^−/−^ mice. (a, b) Representative immunohistochemical images and quantification of VCAM‐1 expression in the aortic root. Scale bar: 200 μm. (c, d) Representative images of TUNEL staining and quantitative analysis. Scale bar: 200 μm. (e, f) Evans blue staining of the mid‐aorta and determination of Evans blue content. Scale bar: 200 μm. Data were presented as the mean ± SEM (*n* = 3). ^###^ *p* < .001 versus Ctrl group; ***p < .001 versus Model group.

Vascular cell adhesion molecule 1 (VCAM‐1) is an essential point in linking pro‐inflammatory activation of endothelial cells to atherosclerotic disease (Cybulsky et al., 2001). Immunofluorescence experiments of aortic showed that VCAM‐1 expression was significantly increased in the Model group, which was improved in HSYA‐treated mice (Figure 4A,B). A significant increase in TUNEL fluorescence intensity at the aortic root in the Model group, while HSYA administration resulted in a significant decrease (Figure 4C,D). It suggested that HSYA reduced the number of apoptotic cells within the aortic plaque and enhanced plaque stability. Next, permeability is an objective measure of vascular endothelial barrier function. Currently, the marker leakage assay is one of the commonly used approaches it can measure endothelial cell permeability by detecting the concentration of large molecule markers, such as Evans blue‐labeled albumin, leaking from the endothelial cells (Guo et al., 2009). To investigate the effect of HSYA on vascular endothelial permeability, we observed and measured the Evans blue content of aortic tissue, and we observed that the HSYA group showed lighter arterial wall color and Evans blue content reduced than the Model group (Figure 4E,F). These findings suggesting that the reduction in the aortic endothelial permeability was associated with the beneficial effects of HSYA.

### 3.5 HSYA ameliorated serum S1P levels and suppressed protein expression of SphK1, S1PR3, RhoA, and ROCK in ApoE ^−/−^ mice

**FIGURE 5:**
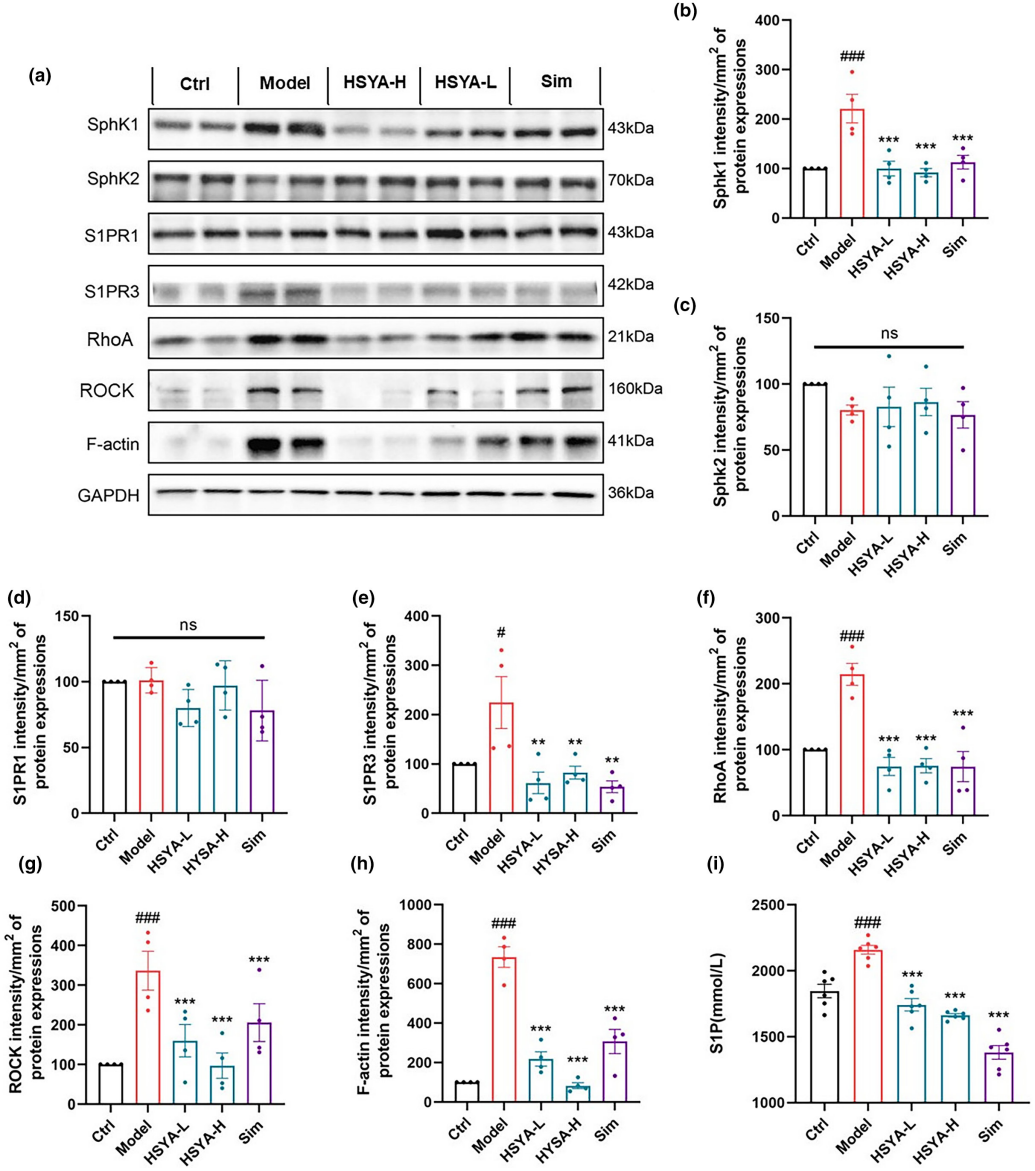
Hydroxysafflor yellow A (HSYA) reduced serum S1P levels and suppressed protein expression of the aortic SphK1/S1P/S1PR3/RhoA/ROCK pathway in ApoE^−/−^ mice. (a–h) Western blot for protein expression of SphK1, SphK2, S1PR1, S1PR3, RhoA, ROCK, and F‐actin in aorta. Data were presented as the mean ± SEM (*n* = 4). ^#^ *p* < .05, ^###^ *p* < .001 versus Ctrl group; **p < .01, ***p < .001 versus Model group. (i) Serum S1P levels. Data were presented as the mean ± SEM (*n* = 6). ^###^ *p* < .001 versus Ctrl group; **p < .01, ***p < .001 versus Model group.

Serum S1P levels and protein expression of SphK1, S1PR3, RhoA, and ROCK in aortic were detected. As shown in Figure 5I, HSYA reduced serum S1P levels. Moreover, HSYA administration resulted in a significant decrease in SphK1, S1PR3, RhoA, ROCK, and F‐actin protein expression. While there was no significant difference in SphK2 and S1PR1 protein expression (Figure 5A–H). Thus, HSYA may inhibit SphK1, reduce S1P production, suppress S1PR3 and RhoA/ROCK protein expression, and then regulate F‐actin expression to improve AS.

### 3.6 HSYA decreased migration and F‐actin expression in vascular endothelial cells

**FIGURE 6:**
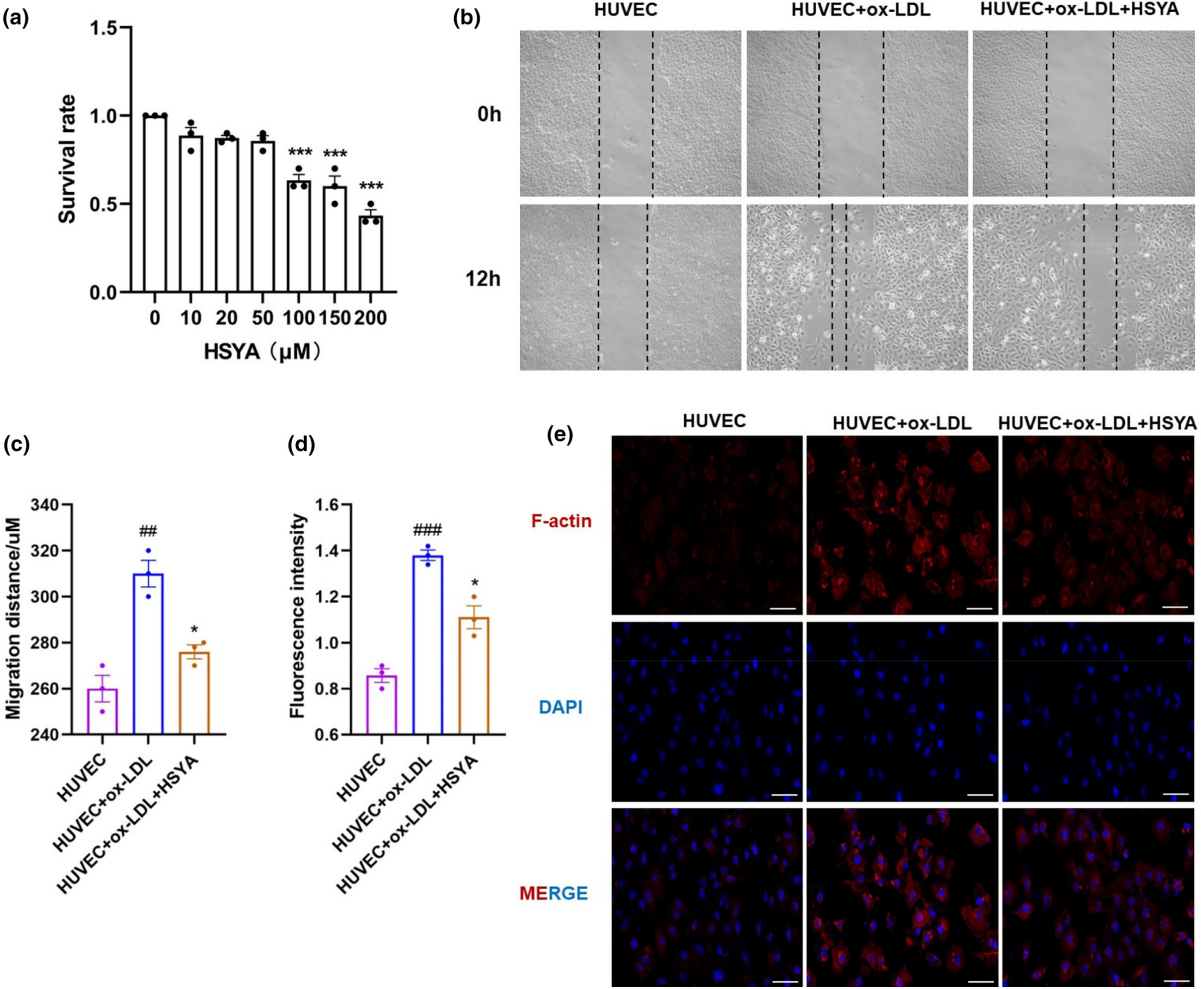
Hydroxysafflor yellow A (HSYA) decreased migration and F‐actin expression in vascular endothelial cells. (a) The cell survival rate of HUVECs. Data were presented as the mean ± SEM (*n* = 3). ***p < .001. All groups were compared to the untreated control group. (b, c) Representative images and quantification of ox‐LDL‐induced vascular endothelial cell migration. Scale bar: 100 μm. Data were presented as the mean ± SEM (*n* = 3). ^##^ *p* < .01 versus HUVEC group; *p < .05 versus HUVEC+ ox‐LDL group. (d, e) Immunofluorescence detection of ox‐LDL‐induced skeletal F‐actin expression. Scale bar: 20 μm. Data were presented as the mean ± SEM (*n* = 3). ^###^ *p* < .001 versus HUVEC group; *p < .05 versus HUVEC+ ox‐LDL group.

Apoptosis is an important toxicological phenomenon, which is a kind of spontaneous and orderly programmed cell death regulated by related genes (Yang et al., 2022). We used CCK‐8 assay to screen the maximum administration dose concentration of HSYA, which is the least toxic to HUVECs, and found that cell viability was significantly decreased when the administration dose concentration was greater than 50 μM (Figure 6A). Therefore, 50 μM was selected as the administration dose and applied to subsequent experiments.

Changes in vascular endothelial cell migration function are also a feature of endothelial impairment. We next established a cell model by ox‐LDL‐induced HUVECs to explore the mechanism of HSYA to improve aortic endothelial permeability. The results revealed the ox‐LDL + HUVEC group significantly migrated toward the center of the scratch, while reduced after HSYA intervention (Figure 6B,C). It is stated that alterations in endothelial cell contractility are the final pathway for different signals and mechanisms leading to changes in permeability, which is mainly influenced by backbone proteins, such as actin and myosin. When subjected to external stimulation such as TNF‐α and platelet activating factor (PAF), F‐actin undergoes reorganization, dense peripheral bundles disappear, and stress fibers are formed, leading to an increase in the size and number of intercellular gaps and an increase in permeability (Wei et al., 2011). So, we also verified by immunofluorescence experiments that the F‐actin was upregulated in the HSYA group (Figure 6D,E). The results described above suggested that HSYA decreased migration and F‐actin expression in HUVECs.

### 3.7 HSYA improved vascular endothelial cell migration and F‐actin expression by inhibiting the SphK1/S1P/S1PR3 pathway

**FIGURE 7:**
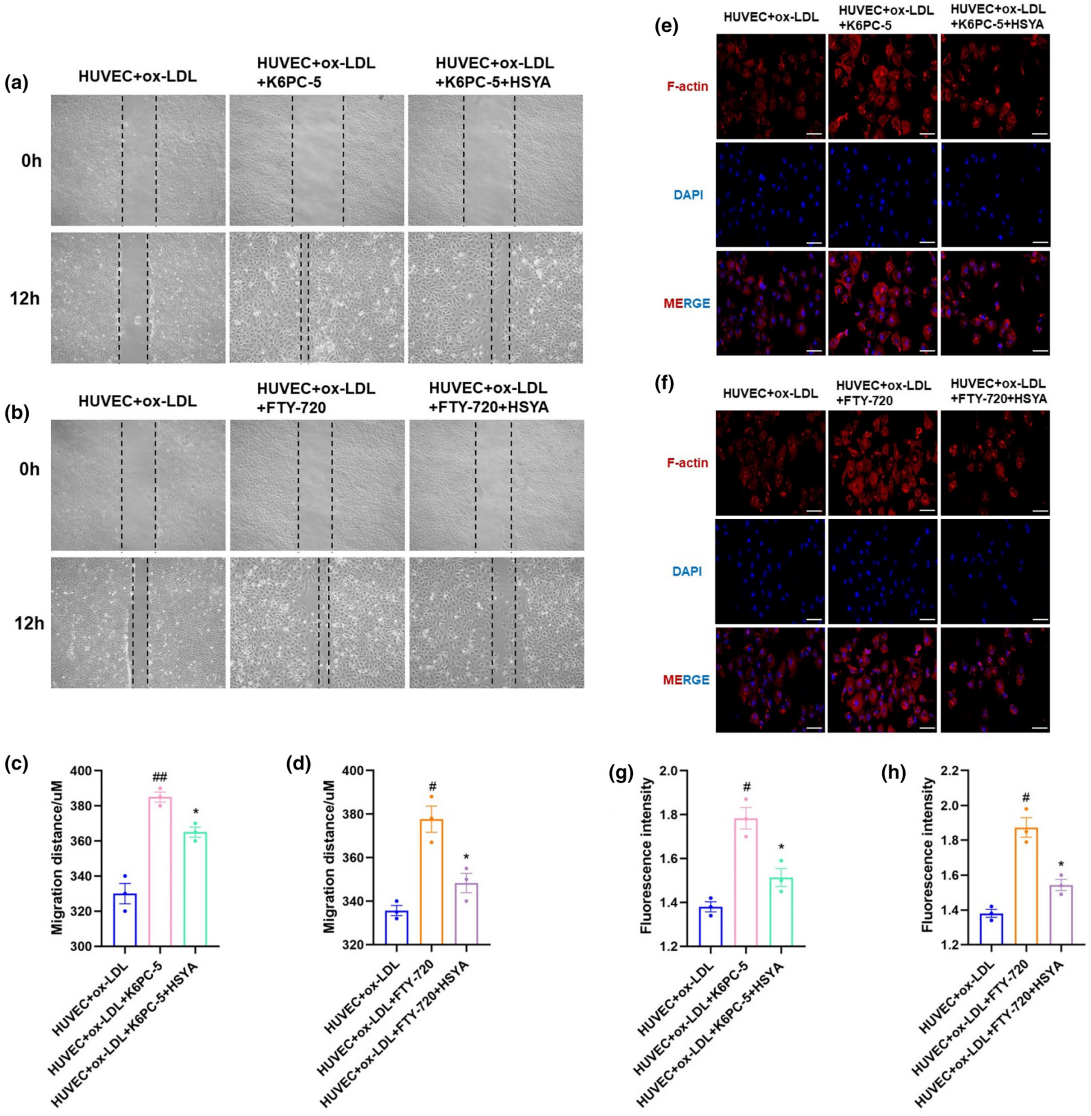
Hydroxysafflor yellow A (HSYA) improved vascular endothelial cell migration and F‐actin expression by inhibiting SphK1/S1P/S1PR. (a, b) Representative images and quantification of ox‐LDL‐induced vascular endothelial cell migration after administration of the SphK1 agonist K6PC‐5 or HSYA intervention. Scale bar: 100 μm. Data were presented as the mean ± SEM (*n* = 3). ^##^ *p* < .01 versus HUVEC+ ox‐LDL group; *p < .05 versus HUVEC+ ox‐LDL + K6PC‐5 group. (c, d) Representative images and quantification of ox‐LDL‐induced vascular endothelial cell migration after administration of the downstream S1P agonist FTY‐720 or HSYA intervention. Data were presented as the mean ± SEM (*n* = 3). ^#^ *p* < .05 versus HUVEC+ ox‐LDL group; *p < .05 versus HUVEC+ ox‐LDL + FTY‐720 group. (e, f) Detection of F‐actin expression by immunofluorescence after administration of the SphK1 agonist K6PC‐5. Scale bar: 20 μm. Data were presented as the mean ± SEM (*n* = 3). ^#^ *p* < .05 versus HUVEC+ ox‐LDL group; *p < .05 versus HUVEC+ ox‐LDL + K6PC‐5 group. (g, h) Detection of F‐actin expression by immunofluorescence after administration of the downstream S1P agonist FTY‐720. Data were presented as the mean ± SEM (*n* = 3). ^#^ *p* < .05 versus HUVEC+ ox‐LDL group; *p < .05 versus HUVEC+ ox‐LDL + FTY‐720 group.

To evaluate whether the therapeutic effect of HSYA was mediated via the SphK1/S1P/S1PR pathway. Hence, we further examined ox‐LDL‐induced HUVEC migration capacity and F‐actin fluorescence intensity after administration of the SphK1 agonist K6PC‐5 and the S1P agonist FTY‐720, respectively. K6PC‐5 is a synthetic derivative of ceramide and a specific activator of SphK1 (Hong et al., 2008). FTY 720 (Fingolimod) is an S1P receptor modulator, which is the pan agonist targeting S1PR1, 3, 4, and 5 (Urbano et al., 2013). It was found that HUVECs significantly migrated toward the center of the scratch after the administration of K6PC‐5 and FTY‐720, respectively. However, the migration distance was blunted after HSYA intervention (Figure 7A–D), which was also confirmed by immunofluorescence experiments, the fluorescence intensity of F‐actin was enhanced in the ox‐LDL + HUVEC+K6PC‐5 and ox‐LDL + HUVEC+FTY‐720 group, which was reversed by HSYA treatment (Figure 7E–H). Thus, it is reasonable to believe that HSYA improved vascular endothelial cell migration and F‐actin expression by inhibiting SphK1/S1P/S1PR pathway.

### 3.8 HSYA inhibits SphK1/S1P/S1PR3/RhoA/ROCK pathway in vitro

**FIGURE 8:**
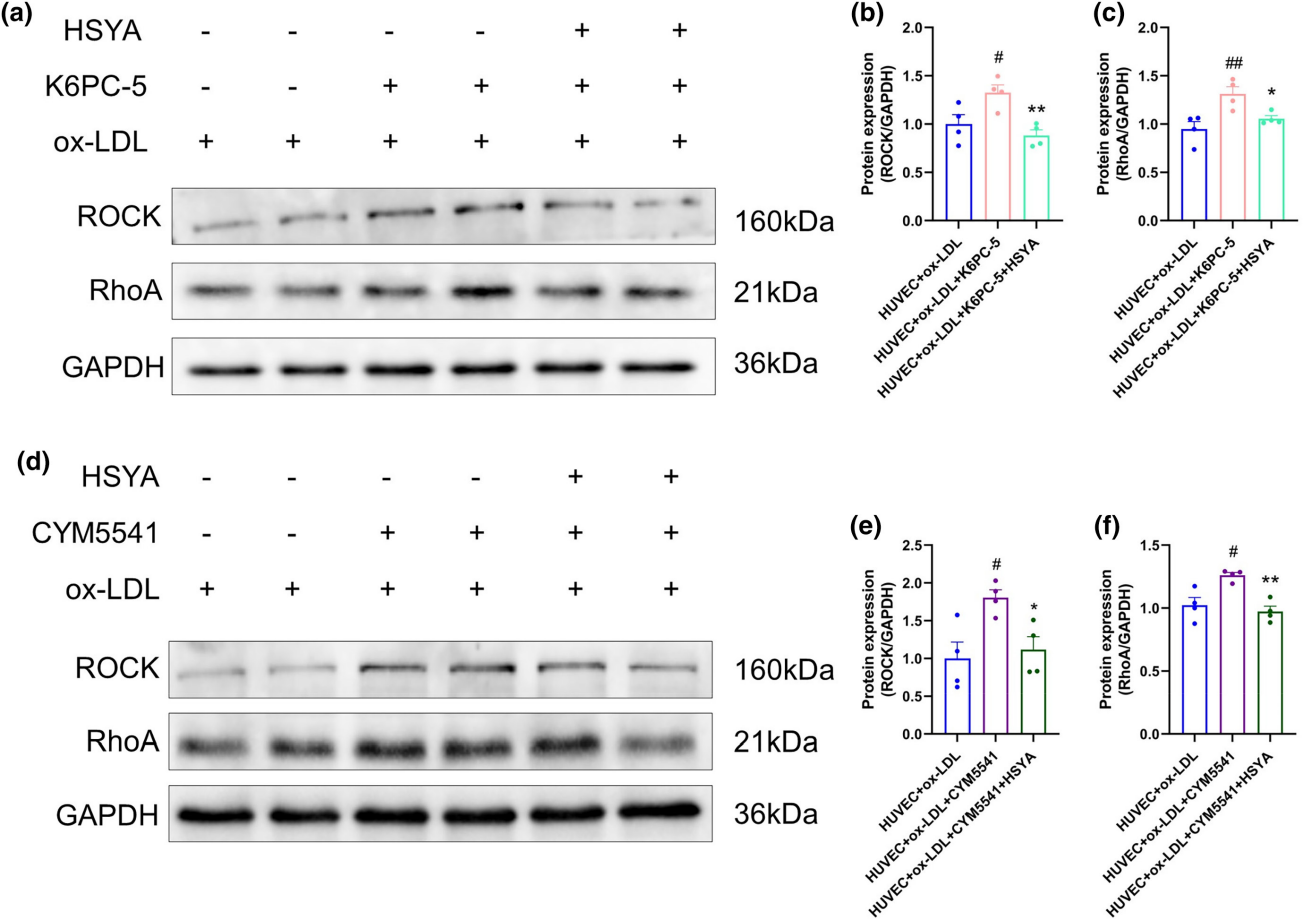
Hydroxysafflor yellow A (HSYA) inhibited SphK1/S1P/S1PR3/RhoA/ROCK pathway in vitro. (a–c) Protein expression of ROCK and RhoA after stimulation of HUVECs by ox‐LDL, SphK1 agonist K6PC‐5, or HSYA was detected by western blotting and quantified by ImageJ software. Data were presented as the mean ± SEM (*n* = 4). ^#^ *p* < .05, ^##^ *p* < .01 versus HUVEC+ ox‐LDL group; *p < .05, **p < .01 versus HUVEC+ ox‐LDL + K6PC‐5 group. (d–f) Protein expression of ROCK and RhoA after stimulation of cellular HUVECs by ox‐LDL, S1PR3 receptor agonist CYM5541, or HSYA was detected by western blotting and quantified by ImageJ software. Data were presented as the mean ± SEM (*n* = 4). ^#^ *p* < .05 versus HUVEC+ ox‐LDL group; **p < .01    versus HUVEC+ ox‐LDL + CYM5541 group.

To further confirm whether the SphK1/S1P/S1PR3/RhoA/ROCK pathway is essential for ox‐LDL‐induced HUVECs after HSYA treatment, HUVECs were first treated with the SphK1 agonist K6PC‐5. Western blotting analyses show that, compared with the ox‐LDL‐induced treatment group, K6PC‐5 significantly enhanced the protein expression of RhoA and ROCK, while HSYA significantly decreased the protein expression of RhoA and ROCK (Figure 8A–C). Next, ox‐LDL‐induced HUVECs were treated with the S1PR3‐selective receptor agonist CYM5541 to further validate the effect of HSYA on AS vascular endothelial permeability after S1PR3 intervention. As shown in Figure 8D–F, CYM5541 enhanced the protein expression levels of RhoA and ROCK. Compared with the HUVEC+ ox‐LDL + CYM5541 group, HSYA significantly reversed these results. This was consistent with our results of in vivo experiments that HSYA inhibited SphK1, reduced the generation of S1P, and inhibited S1PR3 receptor and RhoA/ROCK protein expression.

## 4 DISCUSSION

So far, many studies have shown that HSYA is a natural protective agent that deserves further development (Guo et al., 2023; Xue et al., 2021). In China, injections with HSYA as the main ingredient have achieved safe and good efficacy in clinical studies and observations for the treatment of blood stasis in acute ischemic stroke (Hu et al., 2020). HSYA also has antiplatelet aggregation, antithrombotic, lipid‐lowering, anti‐myocardial infarction, inhibition of cardiomyocyte apoptosis, and anti‐oxidative stress functions (Yang et al., 2020; Zhang et al., 2014). Previous studies of HSYA anti‐AS have focused on the fact that HSYA can inhibit inflammatory responses and thus affect the progression of AS (Feng et al., 2023; Wang et al., 2016). In fact, vascular endothelial dysfunction and increased permeability play an important role in AS (Wakasugi et al., 2024). It has been reported that HSYA inhibits the increase in pulmonary capillary permeability induced by acute lung injury (Liu et al., 2014), crosses the blood–brain barrier (BBB), and reduces the permeability of the blood–brain barrier in traumatic brain injury (Xu et al., 2021). It shows that HSYA has the effect of regulating vascular permeability, but whether it improves AS and its mechanism by regulating the permeability of vascular endothelial cells is still unclear. Here, with HFD‐induced ApoE^−/−^ mice, we demonstrated that HSYA affects AS progression by modulating endothelial permeability.

Endothelial dysfunction is the critical first step in the development of ankylosing spondylitis, and the early stage is characterized by increased endothelial permeability. At this stage, LDL accumulates in the arteries, oxidizes to ox‐LDL, and binds to LOX‐1 (lectin‐like, oxidized low‐density lipoprotein receptor‐1), contributing to endothelial cell activation, increasing permeability, inducing transcription of pro‐inflammatory genes, such as NF‐κB and IL‐6, and activating the expression of E‐selectin and VCAM‐1 (Libby et al., 2011). When permeability is altered, plasma extravasation, aggregation of leukocytes and adhesion factors, and increased uptake of ox‐LDL promote the development of AS (Guo et al., 2018). HSYA has been shown to repair aortic plaque area, lipid deposition, and aortic lesions in AS mice, and to reduce lipids, inflammation, and oxidative stress (Feng et al., 2023; Rong et al., 2023; Xue et al., 2021). This was also confirmed in our current study. Furthermore, we further demonstrated that HSYA also significantly reduced the number of apoptotic cells within aortic plaques and enhanced plaque stability by TUNEL staining experiments. Notably, reduced NO bioavailability, vascular oxidative stress, inflammation, and increased endothelial permeability are all factors that induce endothelial cell dysfunction (Daiber & Chlopicki, 2020). Therefore, we further measured endothelial dysfunction by the primary indicators of endothelium‐derived vasodilatory factors, such as NO and endothelin‐1. NO diffuses from the endothelium into the surrounding vascular smooth muscle cells, causing vasodilation, increased flow, and increased extravasation of fluids and small molecules (Wakasugi et al., 2024). Besides NO, ET‐1, one of the key vasoactive substances produced and secreted by vascular endothelial cells, significantly attenuated endothelium‐dependent vasodilation and mediated changes in NO bioavailability that together “upset” the balance between vasodilation and vasoconstriction (Nishiyama et al., 2017). In the present study, we found that HSYA inhibited serum ET‐1 levels and increased NO content, which indicated that HSYA protects the vascular endothelium and attenuates endothelial damage by increasing NO content. Furthermore, Evans blue content measurements performed on aortic tissue also showed that HSYA decreased vascular permeability and enhanced the vascular barrier. This discovery provides new theoretical support for HSYA as a potential therapeutic agent for AS, particularly significant in protecting endothelial cell function.

What is the intrinsic mechanism by which HSYA improves vascular permeability? It is well established that the SphK/S1P axis represents a key area of interest for cardiovascular scientists, given its relevance to the development and function of the cardiovascular system. SphK1 and SphK2 catalyze the phosphorylation of the lipid sphingosine, resulting in the production of the signal transducer S1P (Cannavo et al., 2017). In our previous study, we found that defects in SphK1 resulted in significant improvements in AS, such as reduced lipid accumulation, inflammatory factors, oxidative stress, aortic plaque area, inflammatory factor infiltration, VCAM‐1 expression, and vascular endothelial permeability (Piao et al., 2024). In the present study, our results showed that aortic SphK1 protein expression was reduced in ApoE^−/−^ mice treated with HSYA, while there was no significant difference in SphK2 protein expression. Notably, S1P receptors are widely expressed in the vascular system and are biologically active phosphorylated lipid growth factors released by activated platelets, which have a variety of physiological activities, such as promoting cell proliferation, preventing apoptosis (Goetzl, 2001), protecting the vascular endothelial barrier (Garcia et al., 2001), and attracting lymphocytes (Mandala et al., 2002), playing an important role in vascular function. Previous studies have shown that high concentrations of S1P promote vascular endothelial cell migration via S1PR1 and S1PR3, whereas inhibition of endothelial cell migration via sphingosine‐1‐phosphate receptor 2 (S1PR2), sphingosine‐1‐phosphate receptor 4 (S1PR4), and sphingosine‐1‐phosphate receptor 5 (S1PR5) has little effect on vascular endothelial cells (Lin et al., 2007; Marsolais & Rosen, 2009). In addition, S1P/S1PR1/S1PR3 axis activation enhances the endothelial barrier and endothelial cell–cell junctions (Gaengel et al., 2012) and promotes vasodilation through eNOS activation (Igarashi et al., 2001), and is therefore thought to have a role in anti‐AS. Interestingly, our previous study has demonstrated that the SphK1/S1P/S1PR3 signaling pathway significantly affects the integrity of the vascular endothelial barrier, thereby attenuating the occurrence of AS (Piao et al., 2024). In the present study, we found that HSYA significantly inhibited aortic S1PR3 protein expression in ApoE^−/−^ mice, while no significant difference was observed in S1PR1 protein expression, suggesting that HSYA may improve AS by inhibiting SphK1, decreasing the production of S1P, inhibiting S1PR3 receptors, and affecting the vascular endothelial barrier.

Ras homolog family member A/Rho‐associated coiled‐coil containing protein kinase (RhoA/ROCK) is downstream of the SphK1/S1P/S1PR signaling pathway, which regulates the formation of stress fibers and focal adhesions and affects the rearrangement of the cytoskeletal protein F‐actin (Zhang et al., 2016). Previous studies have demonstrated that RhoA activation is associated with increased endothelial cell permeability (Breslin et al., 2015; García‐Ponce et al., 2015), disrupting endothelial integrity (Spindler et al., 2010). Furthermore, inhibition of Rho kinase (ROCK), a downstream mediator of RhoA, attenuates S1P‐induced endothelial barrier enhancement (Xu et al., 2007). RhoA and ROCK contribute to endothelial cell dysfunction by remodeling the cytoskeleton, decreasing VE‐calmodulin levels, promoting waveform protein cleavage, and inducing loss of tight junctions (Wei et al., 2017; Yang et al., 2017). In the current study, we found a significant increase in protein expression of aortic RhoA, ROCK, and F‐actin in ApoE^−/−^ mice. However, after administration of HSYA, the protein expression of aortic RhoA, ROCK, and F‐actin was significantly decreased. The results indicate that HSYA may improve AS by inhibiting the SphK1/S1P/S1PR3 pathway, decreasing the protein expression of RhoA/ROCK, and ultimately regulating the expression of F‐actin in the vivo experiments. Our study systematically investigated for the first time the regulatory effects of HSYA on endothelial permeability in an AS model, revealing a novel mechanism for restoring endothelial cell function through inhibition of the inhibitory SphK1/S1P/S1PR3/RhoA/ROCK pathway.

Toward further investigating the intrinsic mechanism of HSYA to ameliorate AS, we mimicked the endothelial damage induced by lipid accumulation in AS by inducing HUVEC with ox‐LDL in our vitro experiments (Bian et al., 2020). Importantly, it has been reported that HSYA partially restores ox‐LDL‐mediated loss of vascular endothelial cell viability and promotes proliferation of damaged vascular endothelial cells (Chen et al., 2019; Xie et al., 2020; Zhang et al., 2022). In our study, we further found that HSYA inhibits ox‐LDL‐induced migration of vascular endothelial cells, thereby affecting vascular endothelial injury and dysfunction. It is important to realize that a key factor in angiogenesis is endothelial cell migration, but when blood vessels are damaged, endothelial cells must migrate to fill the open spaces and restore vascular integrity (Michaelis, 2014). In addition, inhibition of the SphK1/S1P/S1PR signaling pathway suppresses trophoblast migration and inhibits RhoA/ROCK protein activation as with actin polymerization (Liao et al., 2022). In this work, immunofluorescence staining showed that F‐actin rearrangement was increased in ox‐LDL‐stimulated HUVECs, with the appearance of stress fibers, enlarged cellular gaps, paracellular pathway formation, and increased permeability. However, treatment with HSYA improved F‐actin rearrangement. Thus, our findings support that HSYA reduces ox‐LDL‐induced F‐actin rearrangement and increased vascular endothelial permeability.

**FIGURE 9:**
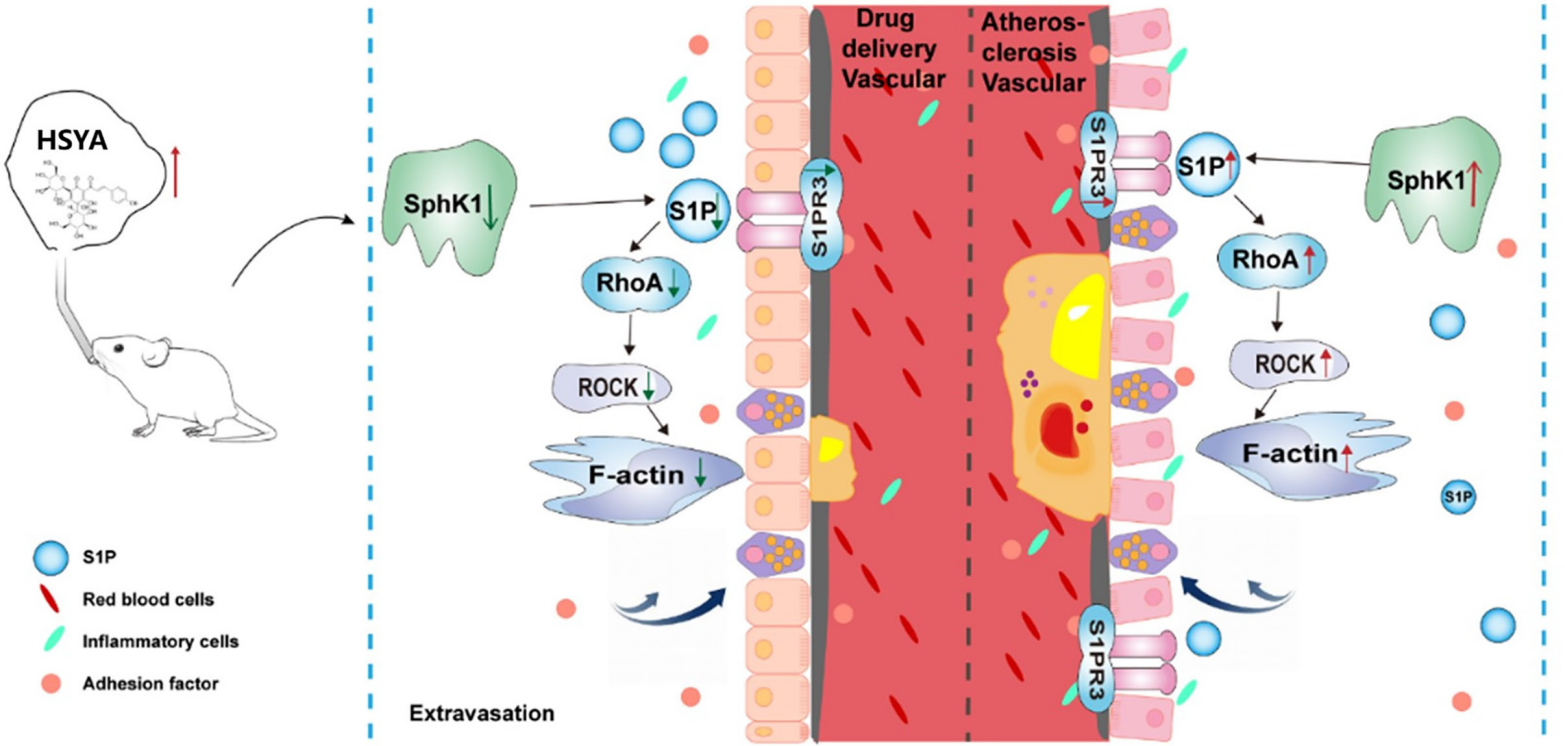
Mechanism of HSYA on AS. HSYA has significant anti‐atherosclerotic effects, and its mechanism may be related to the regulation of HUVECs migration and vascular endothelial cell permeability through the SphK1/S1P/S1PR3/RhoA/ROCK signaling pathway.

To further confirm the mechanism of action of HSYA in regulating vascular endothelial cell permeability through SphK1/S1P/S1PR3, we then stimulated ox‐LDL‐induced HUVEC with the SphK1 agonist K6PC‐5 and the S1P receptor agonist FTY‐720, respectively. We found that in ox‐LDL‐treated HUVECs, activation of SphK1 and S1P enhanced vascular endothelial cell migration and significantly enhanced downstream endothelial cytoskeletal protein F‐actin expression, while HSYA significantly reversed the above results. Then, we stimulated ox‐LDL‐induced HUVEC with the SphK1 agonist K6PC‐5 and the S1PR3 receptor agonist CYM5541, respectively. As expected, protein expression of RhoA/ROCK was significantly enhanced after stimulation of ox‐LDL‐induced HUVEC by K6PC‐5 or CYM5541. However, administration of HSYA treatment significantly inhibited protein expression of RhoA/ROCK. The above results further confirmed that HSYA could inhibit ox‐LDL‐induced endothelial cell migration and improve vascular endothelial cell permeability by regulating the SphK1/S1P/S1PR3/RhoA/ROCK signaling pathway (Figure 9).

Our study also has some limitations. Due to the complexity of the pathogenesis and development of AS, although the present study found that HSYA could improve AS by inhibiting the aortic SphK1/S1P/S1PR3 pathway, downregulating RhoA/ROCK protein expression, affecting downstream F‐actin expression, resulting in decrease of vascular endothelial cell migration ability and vascular endothelial permeability, whether HSYA can improve the AS process by influencing other aspects remains to be further explored.

## 5 CONCLUSIONS

In the present study, we revealed the therapeutic effects of HSYA in AS mice, as indicated by the improved levels of lipids, S1P, inflammatory factors, oxidative stress, and endothelial damage, decreased the aortic lipid droplet area, necrotic core area, and apoptosis in the aortic root, increased the content of elastin fibers and collagen fibers, and that the mechanism of action was related to inhibition of the SphK1/S1P/S1PR3 signaling pathway. These results offered new evidence for the SphK1/S1P/S1PR3/RhoA/ROCK signaling pathway in AS and suggested that HSYA could be used as an effective therapeutic agent or functional food in AS.

## AUTHOR CONTRIBUTIONS

**Yuqing Liu:** Conceptualization (equal); methodology (equal); writing – original draft (equal); writing – review and editing (equal). **Jingyu Piao:** Conceptualization (equal); methodology (equal); writing – original draft (equal); writing – review and editing (equal). **Jiqian He:** Writing – review and editing (supporting). **Zhuoxuan Su:** Writing – review and editing (supporting). **Zhizhong Luo:** Writing – review and editing (supporting). **Duosheng Luo:** Methodology (lead); writing – review and editing (lead).

## FUNDING INFORMATION

This work was supported by grants from the National Natural Science Foundation of China (81830113, 82074210), Major Basic and Applied Basic Research Projects of Guangdong Province of China (2019B030302005), and the National Key R&D Program of China (2023YFC3606200).

## CONFLICT OF INTEREST STATEMENT

There are no conflicts of interest to declare.

## ETHICS STATEMENT

This study was approved by the Experimental Animal Ethics Committee of Guangdong Pharmaceutical University (Ethics Committee No. GDPULAC2020059).

## Data Availability

The data that support the findings of this study are available from the corresponding author upon reasonable request.
